# A Case of Severe Fever With Thrombocytopenia Syndrome Co-infected With Pseudomonas aeruginosa and Aspergillus fumigatus

**DOI:** 10.7759/cureus.98752

**Published:** 2025-12-08

**Authors:** Jing Zhao, Yanjun Wang, Huan Zhang, Jing Feng

**Affiliations:** 1 Department of Infectious Diseases, Weifang People's Hospital, Weifang City, CHN

**Keywords:** aspergillus fumigatus, metagenomic next-generation sequencing, mortality, pseudomonas aeruginosa, severe fever with thrombocytopenia syndrome

## Abstract

Severe fever with thrombocytopenia syndrome (SFTS) is a tick-borne infectious disease with a high mortality rate. Co-infections with SFTS virus (SFTSV) and other pathogens can exacerbate the condition, leading to rapidly progressive multiple organ failure. We reported a case of SFTS complicated by *Pseudomonas aeruginosa* and *Aspergillus fumigatus*. Despite active symptomatic supportive treatment (including anti-virus, anti-infection, anti-antifungal treatment, liver protection, and other advanced life supports), the patient's condition deteriorated rapidly, leading to multiple organ failure. The patient was discharged home and died on the same day. The next day, her blood test results reported that SFTSV, *Pseudomonas aeruginosa* and *Aspergillus fumigatus* were detected through metagenomic next-generation sequencing (mNGS). It suggests that early pathogen screening and targeted anti-infective treatment are crucial for improving the prognosis.

## Introduction

Severe fever with thrombocytopenia syndrome (SFTS), an emerging tick-borne disease caused by the SFTS bunyavirus (SFTSV), has posed a significant public health threat in Asia since its identification in China in 2009 [1]. Characterized by fever, thrombocytopenia, and a high case-fatality rate (2.7-45.7%), SFTS can progress rapidly to multi-organ dysfunction syndrome (MODS) [2,3]. While invasive fungal infections, particularly aspergillosis, have been increasingly recognized as fatal complications in severe SFTS cases [4,5], concurrent bacterial and fungal co-infections remain sparsely reported. Such polymicrobial challenges could obscure the clinical picture, accelerate clinical decline, and demand a nuanced diagnostic approach. Here, we reported a fatal case of SFTS complicated by *Pseudomonas aeruginosa* and *Aspergillus fumigatus*.

## Case presentation

A 53-year-old woman from Shandong Province, with medical history of hypertension and type 2 diabetes, visited a local clinic on July 8th, 2023, with a complaint of fever for four days. Her body temperature was 38.3°C accompanied by chills, myalgia and fatigue, and nausea. Initial treatment with intravenous cefoxitin and oral nimesulide was ineffective. Later that same day, she developed acute neurological deterioration including agitation, delirium, and incoherent speech, prompting hospital admission.

Initial assessment and differential diagnosis

On admission to the local hospital, key findings included leukocytopenia (white blood cell (WBC) 1.07×10⁹/L), thrombocytopenia (platelet (PLT) 38×10⁹/L), mild liver injury (alanine aminotransferase (ALT) 57 U/L, aspartate aminotransferase (AST) 139U/L) and myocardial injury (lactate dehydrogenase (LDH) 1050U/L). ECG showed a prolonged QT interval. Initial cranial and chest CT scans were unremarkable. The acute onset of fever, thrombocytopenia, leukopenia, and neurological symptoms in a rural resident during summer prompted a broad differential diagnosis. Primary considerations included: SFTS, other viral infections (such as hemorrhagic fever with renal syndrome), rickettsial diseases, systemic bacterial infections (particularly sepsis), and non-infectious etiologies including autoimmune disorders (e.g., systemic lupus erythematosus, thrombotic thrombocytopenic purpura), adverse drug reactions, and primary hematological diseases. The absence of meningeal signs (neck stiffness, Kernig's sign) made typical bacterial meningitis less likely but did not rule out encephalitis. The lack of a recalled tick bite or pet exposure, while common in many SFTS cases, did not alter the high initial suspicion for SFTS in an endemic area, as exposure history is often absent. Treatment with methylprednisolone, leucogen, and umibeogin was given, then she was referred to the Department of Infectious Diseases, WeiFang People’s Hospital.

Hospital course and management

**Figure 1:**
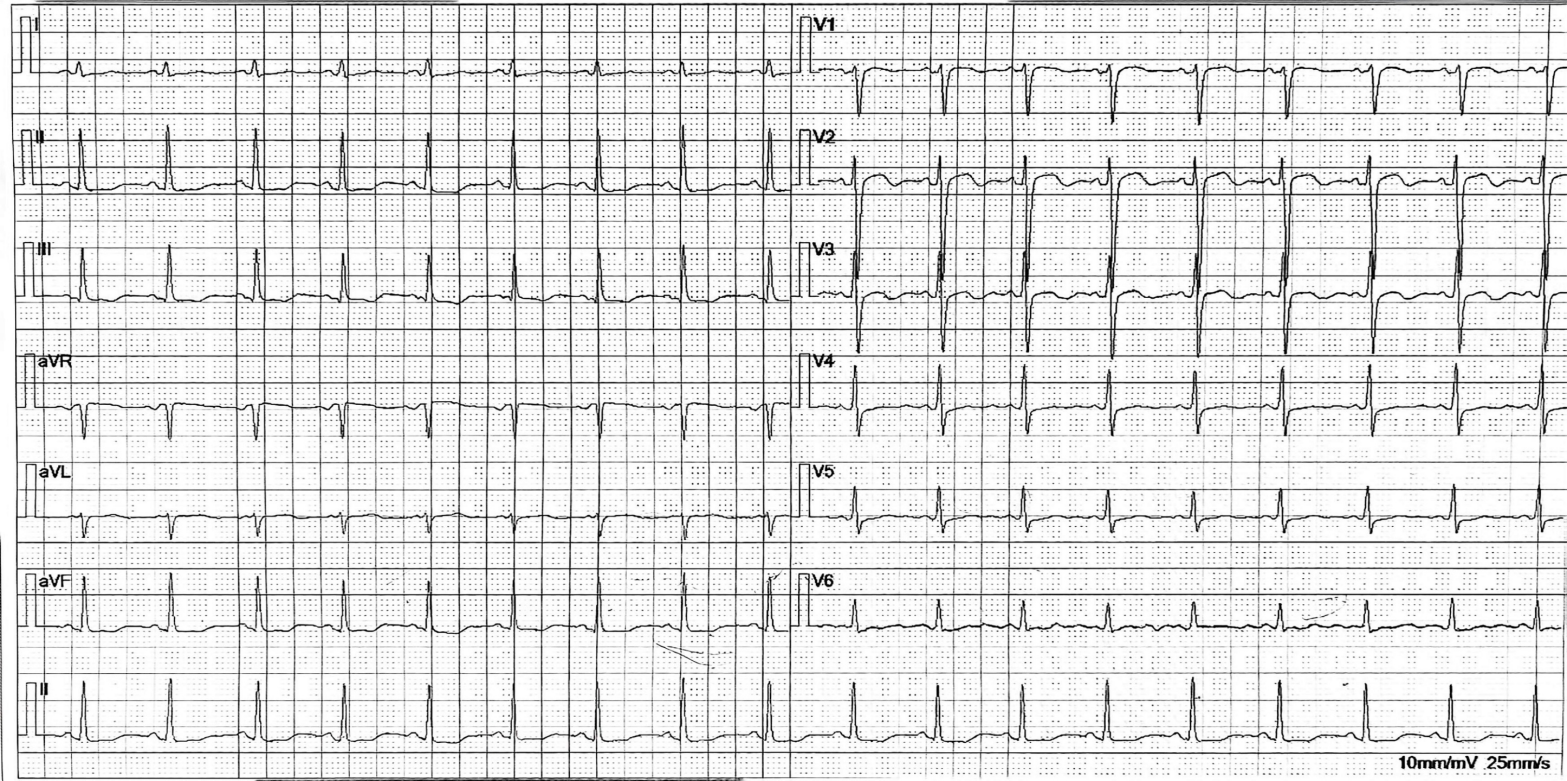
Electrocardiogram at admission to our hospital The ECG demonstrates non-specific ST-T wave abnormalities (flattened T waves in leads II, III, aVF, V4-V6). These findings are consistent with a non-specific myocardial insult, potentially due to viral myocarditis, cytokine-mediated injury, or electrolyte imbalance commonly seen in severe systemic infections like severe fever with thrombocytopenia syndrome (SFTS).

On admission, her Glasgow Coma Scale was E4V3M5. Vital signs showed tachycardia and hypotension. Physical examination revealed no petechiae, purpura, meningeal signs, or eschars. We retrieved the following information: she had no travel history and there were no pets in her family. Laboratory investigations confirmed and extended the initial findings (Table 1). ECG showed ST-T changes (Figure 1). Marked thrombocytopenia (PLT 46×10⁹/L), significantly elevated inflammatory markers (procalcitonin (PCT) 6.68ng/mL, IL-6 35.68 pg/mL, ferritin >1650ng/mL), worsening liver function (AST 286U/L), and coagulopathy (elevated activated partial thromboplastin time (APPT) and D-dimer) reinforced the likelihood of severe SFTS with evolving MODS. Empirical treatment was initiated immediately, comprising intravenous ribavirin (500mg every 12 hours), meropenem (1g every eight hours) for broad-spectrum bacterial coverage, methylprednisolone (40 mg every 12 hours) to mitigate potential cytokine storm, along with supportive care (platelet transfusion, liver protection, glycemic control, and blood pressure control).

**Table 1:** Key laboratory results at admission to our hospital MAS: macrophage activation syndrome, DIC: disseminated intravascular coagulation

Laboratory tests	Results	Reference range	Clinical implication
Hematology			
White blood cells (/L)	1.8×10^9^	3.5 - 9.5×10^9^	Severe leukopenia
Hemoglobin (g/L)	121	115 - 150	Normal hemoglobin
Platelet count (/L)	46×10^9^	125 - 350×10^9^	Severe thrombocytopenia
Inflammatory Markers			
C-reactive protein (mg/L)	15.9	0 - 4	Moderate elevation
Procalcitonin (ng/mL)	6.68	0 - 0.5	Highly suggestive of bacterial co-infection
Interleukin (IL)-6 (pg/mL)	35.68	0 - 7	Marked inflammatory response
Ferritin level (ng/mL)	﹥1,650	10 - 291	Extreme elevation, suggests cytokine storm/MAS
Liver Function			
Alanine aminotransferase (U/L)	85	0 - 40	Mild hepatocytolysis
Aspartate aminotransferase (U/L)	286	0 - 35	Moderate hepatocytolysis
Cardiac & Muscle			
Lactate dehydrogenase (U/L)	1,450	120 - 250	Marked elevation, indicates tissue damage
Coagulation			
Activated partial thromboplastin time (S)	44.4	22 - 38	Prolonged coagulopathy
D-Dimer (ug/mL)	15.3	0 - 1	Severely elevated, suggests DIC

Diagnostic refinement and clinical deterioration

Despite treatment, her condition worsened within hours, with persistent high fever, progressive agitation leading to generalized convulsions, coma, and hypoxemia requiring emergent endotracheal intubation and mechanical ventilation. Neurology and ICU consultations were obtained. Management was escalated to include intravenous immunoglobulin (15g/d for three days), vasopressor support for refractory hypotension. Serial laboratory monitoring revealed progressive multi-organ failure: acute kidney injury (creatinine rising to 112 µmol/L), worsening liver function and coagulopathy, persistently elevated cardiac enzymes and inflammation markers levels. A critical turning point was the preliminary report of *Pseudomonas aeruginosa* growth in blood cultures on hospital day two. Accordingly, antimicrobial therapy was streamlined to high-dose meropenem (2g every eight hours, extended infusion time) based on local susceptibility patterns, though formal susceptibility testing was pending. The patient was then transferred to the ICU for PLT, fresh-frozen plasma, cryoprecipitate transfusion and advanced life support. The patient's condition deteriorated rapidly, with decreasing blood pressure and oxygen saturation. A lumbar puncture performed under platelet transfusion revealed findings suggestive of meningoencephalitis (cerebrospinal fluid pressure 170mmH₂O, elevated protein 3757.9 mg/L, 26×106/L nucleated cells, 96% monocytes, strongly positive Pandy protein test), but CSF bacterial and fungal cultures remained negative. Simultaneously, an elevated serum (1→3)-β-D-glucan level (229.7 pg/mL on day three) was observed.

Outcome and post-mortem diagnosis

Given the grave prognosis and rapid deterioration despite maximal support, the family discharged her home on hospital day four. The patient died at home on the same day. The next day, metagenomic next-generation sequencing (mNGS) analysis of a blood sample drawn prior to discharge returned, identifying sequences for SFTSV, *Pseudomonas aeruginosa* and *Aspergillus fumigatus* (Table 2), confirming the triple infection.

**Table 2:** Metagenomic next-generation sequencing results from blood Metagenomic next-generation sequencing (mNGS) was performed on plasma. The "Positive Reference Threshold" is based on the laboratory's validated criteria, where the detection of any specific sequence for Aspergillus spp. in blood is considered clinically significant for invasive disease, and sequences for typical bacterial pathogens like P. aeruginosa above a low threshold (e.g., ≥8) are indicative of true infection rather than background noise or contamination. SFTS: severe fever with thrombocytopenia syndrome, SFTSV: SFTS virus

Detection indicators	Number of sequences	Positive reference range	Interpretation
SFTS bunyavirus	4,061	≥3	Confirms active SFTSV infection
Pseudomonas aeruginosa	16	≥8	Supports blood culture finding, indicates bacteremia
Aspergillus fumigatus	33	≥8	Highly suggestive of invasive fungal infection (probable invasive aspergillosis)

## Discussion

SFTS is an acute zoonotic disease primarily affecting adults, although cases in children have also been reported previously [5]. Moreover, re-infection cases have been found [6]. A minority of patients develop severe illness, progressing rapidly to critical or severe conditions, which can ultimately lead to death due to multiple organ failure. However, SFTS with co-infections were more likely to have neurological symptoms and higher mortality rates, which require early diagnosis and effective therapeutic strategies [7].

The case reported a fatal case of SFTS complicated by concurrent* Pseudomonas aeruginosa* bacteremia and probable invasive aspergillosis. Co-infections including pulmonary infection, bloodstream infection, and abdominal infection were observed in SFTS patients from China [8].

The pathogens of bloodstream co-infections include *Enterococcus faecalis*, *Escherichia coli*, and *Staphylococcus epidermidis* [8,9]. In our patient, both conventional blood cultures and mNGS identified *Pseudomonas aeruginosa*. It exemplifies a critical clinical scenario where viral-induced immunosuppression sets the stage for opportunistic invaders, potentially leading to a synergistic and overwhelming infection. In addition, evidence suggests a possible link between *Pseudomonas* infection and tick exposure, as *Pseudomonas* species have been detected in Chinese ticks [10]. So, *Pseudomonas aeruginosa* bacteremia in SFTS may arise from either the immunocompromised state secondary to the viral infection or direct inoculation via a tick bite.

In this case, conventional diagnostics (blood culture) identified *Pseudomonas aeruginosa*, allowing for targeted antibiotic escalation. Although the significantly elevated β-D-glucan level and the later mNGS results collectively suggested an invasive fungal infection (*Aspergillus fumigatus*), targeted antifungal therapy could not be initiated during the hospitalization. The confirmatory mNGS report identifying *Aspergillus fumigatus* was only available after the patient had left the hospital. If this result can be obtained in a timely manner, the standard treatment strategy should be to immediately initiate potent antifungal therapy.

The diagnosis of aspergillosis remained presumptive based on clinical context and biomarker (β-D-glucan) until post-mortem mNGS confirmation. SFTSV and Aspergillus con-infection are commonly observed in severe and critical cases [4,6,11,12]. Notably, many patients lack traditional high-risk factors for fungal infections, such as corticosteroid use or prolonged broad-spectrum antibiotic therapy. Intriguingly, Aspergillus has been detected in the heads of hard ticks [13], and Aspergillus fumigatus sequences have been identified in the peripheral blood of patients during the early stages of SFTSV infection through mNGS [14]. This raises the possibility that tick bites could lead to direct entry of Aspergillus into the bloodstream.

At present, the diagnostic methods mainly include microbial culture and microscopic examination. Advanced techniques like mNGS and targeted NGS (tNGS) have proven valuable in diagnosing rare or polymicrobial infections, particularly in culture-negative cases. Rather than advocating for universal application, we recommend a risk-stratified diagnostic strategy. On the premise of clinical suspicion of co-infections, it is necessary to select appropriate means such as culture and NGS according to the actual situation to make a rapid and accurate diagnosis and guide timely intervention.

## Conclusions

This case offered a complex co-infections of bacterium, fungus and virus, including *Pseudomonas aeruginosa* and *Aspergillus fumigatus*. Medical personnel should be highly vigilant for co-infections in SFTS patients. Early and aggressive diagnostic efforts, including the strategic use of mNGS in complex cases, are paramount to identify these pathogens in time to institute potentially life-saving targeted therapy. A proactive, individualized approach to pathogen surveillance in high-risk SFTS patients is essential to improve outcomes.
